# Supplementary material for: Multi‐dataset identification of innovative feature genes and molecular mechanisms in keratoconus
Source: J Cell Mol Med. 2024 Sep 19;28(18):e70079. doi: 10.1111/jcmm.70079 (PMC11412914; doi:10.1111/jcmm.70079)
Supplement: Supplementary file 2 — Material S2. [file JCMM-28-e70079-s002.zip › 7.GSEA analysis/6.SNORA74B/KEGG_GLYCOSAMINOGLYCAN_BIOSYNTHESIS_KERATAN_SULFATE.html]

Details for gene set KEGG\_GLYCOSAMINOGLYCAN\_BIOSYNTHESIS\_KERATAN\_SULFATE[GSEA]

|  || Dataset | SNORA74B.group.cls#h\_versus\_l |
| Phenotype | group.cls#h\_versus\_l |
| Upregulated in class | l |
| GeneSet | KEGG\_GLYCOSAMINOGLYCAN\_BIOSYNTHESIS\_KERATAN\_SULFATE |
| Enrichment Score (ES) | -0.48050395 |
| Normalized Enrichment Score (NES) | -1.3498145 |
| Nominal p-value | 0.14313726 |
| FDR q-value | 0.31469274 |
| FWER p-Value | 0.931 |
Table: GSEA Results Summary

  

Fig 1: Enrichment plot: KEGG\_GLYCOSAMINOGLYCAN\_BIOSYNTHESIS\_KERATAN\_SULFATE      
 Profile of the Running ES Score & Positions of GeneSet Members on the Rank Ordered List

  

| SYMBOL | TITLE | RANK IN GENE LIST | RANK METRIC SCORE | RUNNING ES | CORE ENRICHMENT || 1 | CHST2 | na | 918 | 0.502 | 0.1440 | No |
| 2 | B4GALT2 | na | 10010 | 0.092 | -0.0866 | No |
| 3 | CHST6 | na | 10052 | 0.091 | -0.0567 | No |
| 4 | ST3GAL1 | na | 13468 | 0.042 | -0.1408 | No |
| 5 | B4GAT1 | na | 18322 | -0.010 | -0.2773 | No |
| 6 | B3GNT7 | na | 18460 | -0.011 | -0.2774 | No |
| 7 | B4GALT1 | na | 20837 | -0.038 | -0.3329 | No |
| 8 | B4GALT4 | na | 24861 | -0.096 | -0.4163 | No |
| 9 | B4GALT3 | na | 27091 | -0.141 | -0.4325 | Yes |
| 10 | B3GNT2 | na | 27484 | -0.151 | -0.3927 | Yes |
| 11 | CHST4 | na | 27488 | -0.151 | -0.3416 | Yes |
| 12 | ST3GAL2 | na | 31591 | -0.300 | -0.3579 | Yes |
| 13 | CHST1 | na | 32108 | -0.328 | -0.2614 | Yes |
| 14 | FUT8 | na | 33139 | -0.408 | -0.1528 | Yes |
| 15 | ST3GAL3 | na | 34214 | -0.585 | 0.0148 | Yes |
Table: GSEA details [plain text format]

  

Fig 2: KEGG\_GLYCOSAMINOGLYCAN\_BIOSYNTHESIS\_KERATAN\_SULFATE      
 Blue-Pink O' Gram in the Space of the Analyzed GeneSet

  

Fig 3: KEGG\_GLYCOSAMINOGLYCAN\_BIOSYNTHESIS\_KERATAN\_SULFATE: Random ES distribution      
 Gene set null distribution of ES for **KEGG\_GLYCOSAMINOGLYCAN\_BIOSYNTHESIS\_KERATAN\_SULFATE**
